# IIIDB: a database for isoform-isoform interactions and isoform network modules

**DOI:** 10.1186/1471-2164-16-S2-S10

**Published:** 2015-01-21

**Authors:** Yu-Ting Tseng, Wenyuan Li, Ching-Hsien Chen, Shihua Zhang, Jeremy JW Chen, Xianghong Jasmine Zhou, Chun-Chi Liu

**Affiliations:** 1 Institute of Genomics and Bioinformatics, National Chung Hsing University, Taiwan; 2 Molecular and Computational Biology, University of Southern California, USA; 3 Department of Internal Medicine, Division of Pulmonary and Critical Care Medicine and Center for Comparative Respira-tory Biology and Medicine, University of California Davis, Davis, California, USA; 4 National Center for Mathematics and Interdisciplinary Sciences, Academy of Mathematics and Systems Science, CAS, Bei-jing, China; 5 Institute of Biomedical Sciences, National Chung Hsing University, Taiwan; 6 Institute of Molecular Biology, National Chung Hsing University, Taiwan; 7 Agricultural Biotechnology Center, National Chung Hsing University, Taiwan

## Abstract

**Background:**

Protein-protein interactions (PPIs) are key to understanding diverse cellular processes and disease mechanisms. However, current PPI databases only provide low-resolution knowledge of PPIs, in the sense that "proteins" of currently known PPIs generally refer to "genes." It is known that alternative splicing often impacts PPI by either directly affecting protein interacting domains, or by indirectly impacting other domains, which, in turn, impacts the PPI binding. Thus, proteins translated from different isoforms of the same gene can have different interaction partners.

**Results:**

Due to the limitations of current experimental capacities, little data is available for PPIs at the resolution of isoforms, although such high-resolution data is crucial to map pathways and to understand protein functions. In fact, alternative splicing can often change the internal structure of a pathway by rearranging specific PPIs. To fill the gap, we systematically predicted genome-wide isoform-isoform interactions (IIIs) using RNA-seq datasets, domain-domain interaction and PPIs. Furthermore, we constructed an III database (IIIDB) that is a resource for studying PPIs at isoform resolution. To discover functional modules in the III network, we performed III network clustering, and then obtained 1025 isoform modules. To evaluate the module functionality, we performed the GO/pathway enrichment analysis for each isoform module.

**Conclusions:**

The IIIDB provides predictions of human protein-protein interactions at the high resolution of transcript isoforms that can facilitate detailed understanding of protein functions and biological pathways. The web interface allows users to search for IIIs or III network modules. The IIIDB is freely available at http://syslab.nchu.edu.tw/IIIDB.

## Background

Protein-protein interactions (PPIs) perform and regulate fundamental cellular processes. As a consequence, identifying interacting partners for a protein is essential to understand its functions. In recent years, remarkable progress has been made in the annotation of all functional interactions among proteins in the cell. However, in both experimentally derived and computationally predicted protein-protein interactions, a "protein" generally refers to "all isoforms of the respective gene." Yet, it is known that alternative splicing often impacts PPI by either directly affecting protein interacting domains, or by indirectly impacting other domains, which, in turn, impact the PPI binding [1]. That is, alternative splicing can modulate the PPIs by altering the protein structures and the domain compositions, leading to the gain or loss of specific molecular interactions that could be key links of pathways (reviewed in reference [2]). It is very likely that different isoforms of the same protein interact with different proteins, thus exerting different functional roles. For example, the protein BCL2L1 is alternatively spliced into two isoforms: Bcl-xL (long form) and Bcl-xS (short form) [3], in which Bcl-xL inhibits apoptosis whereas Bcl-xS promotes apoptosis [4]. Vogler et al. reported that the interaction of Bcl-xL and BAK1 in platelets ensures platelet survival [5]. Therefore, comprehensively identifying protein-protein interactions at the isoform level is important to systematically dissect cellular roles of proteins, to elucidate the exact composition of protein complexes, and to gain insights into metabolic pathways and a wide range of direct and indirect regulatory interactions.

Thus far, a series of studies have systematically predicted PPIs [6-10] and established PPI databases, e.g., OPHID [11], POINT [12], STRING [10] and PIPs [7]. With the exception that the IntAct database [13] contains 116 human PPIs with isoform specification, currently, none of those PPI databases has isoform-level PPI data. This is a huge knowledge gap yet to be filled. The rapid accumulation of RNA-seq data provides unprecedented opportunities to study the structures and topological dynamics of PPI networks at the isoform resolution. RNA-seq data provides two unique informative sources for Isoform-Isoform Interaction (III) reconstruction: the absence or presence of specific isoforms under specific conditions, and the co-expression of two isoforms that may contribute to their interaction propensity. In this study, we seize this opportunity to comprehensively predict the possible interactions between splicing isoforms by integrating a series of RNA-seq data with domain-domain interaction data and PPI database. The resulting III network presents a high-resolution map of PPIs, which could be invaluable in studying biological processes and understanding cellular functions.

**Figure 1:**
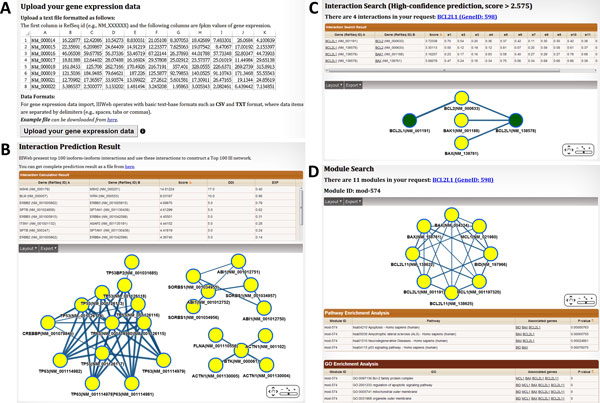
**The screenshots of the isoform interaction and module search function in the IIIDB**. **(A) **Users can upload their own gene expression data for III prediction. **(B) **IIIDB shows top 100 IIIs and uses these interactions to construct an III network. Users can download complete prediction result as a file. **(C) **In interaction search function, users can input a gene symbol or gene ID to search associated IIIs and isoform modules. The interaction search section provides interface on searching high-confidence (score > 2.575) or low-confidence (score > 1.692) prediction. The resulting III table for interaction search shows full evidence values including Pearson correlations for 19 RNA-seq datasets and domain interaction score. **(D) **The resulting page for module search includes the user friendly network graph and pathway/GO enrichment analysis result.

In this report, we described a database, IIIDB, for accessing and managing predicted human IIIs. In the IIIDB, users can differentiate access high-confidence and low-confidence predictions of human IIIs (see detailed description in Result section), and then see the full evidence values for each predicted III. Figure 1 shows the IIIDB web interface screen-shots of the III search and isoform module search function in the IIIDB. Users can upload their won gene expression data for III prediction, and then the users can download the predicted result. The searching function has three major parts: high-confidence interaction prediction search, low-confidence interaction prediction search, and isoform module search. The IIIDB provides auto-complete function in all search functions. Users can input a gene symbol or gene ID in the auto-complete field which provides an interface to quickly find and select matched values.

The IIIDB allows users to easily search IIIs and isoform modules, and then provides the evidence that led to each III prediction. To visualize the interactions with the isoforms of the input gene, we integrated CytoscapeWeb [14] to generate the interactive web-based III network (Figure 1B). Interestingly, the different isoforms within the same gene can be involved with different isoform modules that may open a new door to study differential functionality of isoforms of the gene. The IIIDB also provided GO/pathway enrichment analysis results for each isoform module, which helps biologists to study the biological insights of network modules at isoform level.

## Results

**Figure 2:**
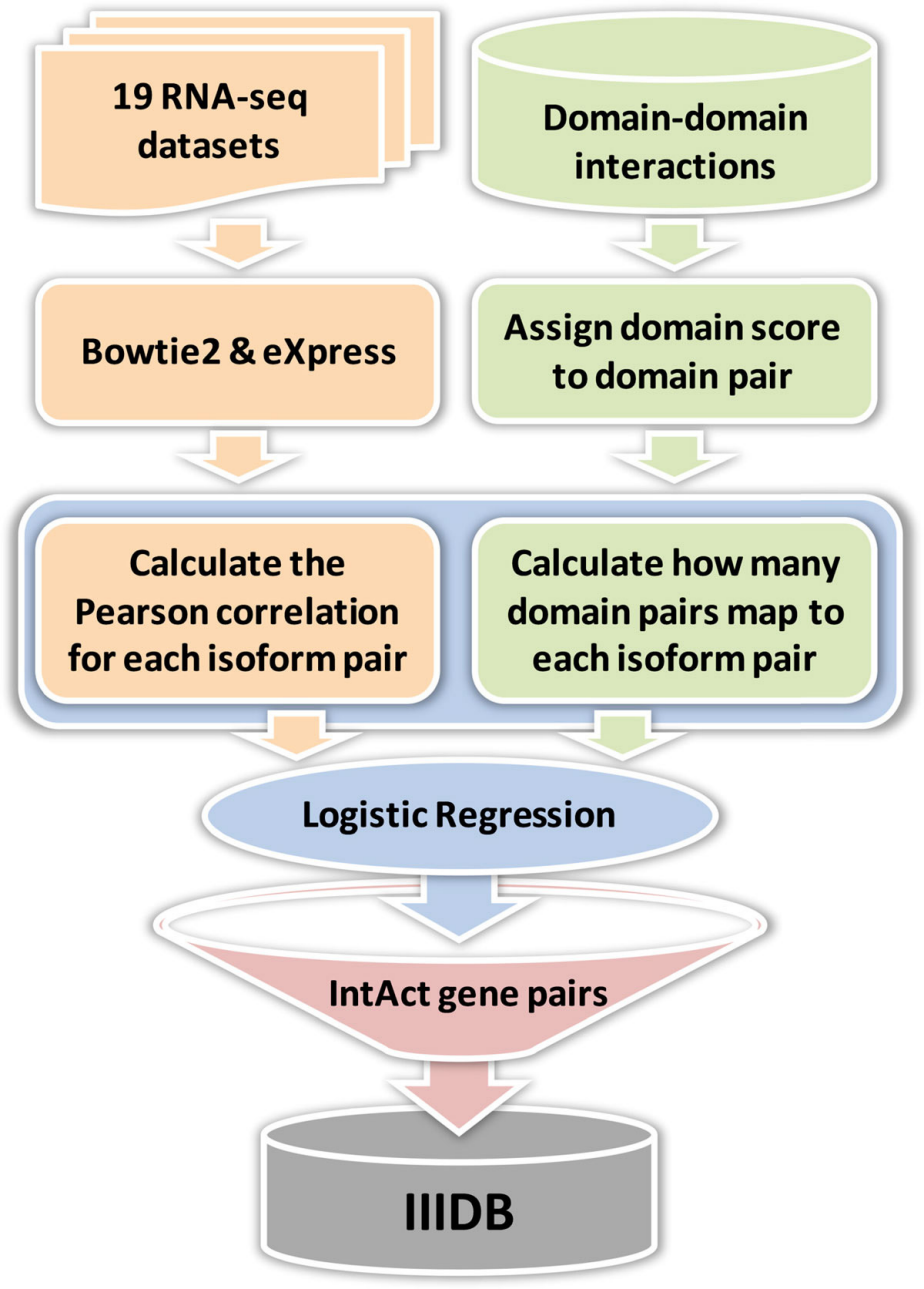
**We performed logistic regression with 19 RNA-seq datasets and the domain-domain interaction database to construct IIIDB**. In RNA-seq data processing, Bowtie2 and eXpress were used to calculate isoform expressions. To confirm with PPI, given an III prediction I1 and I2, we only keep this isoform interaction if the gene symbols of I1 and I2 have PPI in IntAct database.

To obtain the isoform annotations in the human genome, we used the NCBI Reference Sequences (RefSeq) mRNAs as transcriptome annotation [15]. Figure 2 shows the framework of III prediction. We employed the logistic regression approach with 19 RNA-seq datasets (Table 1) and the domain-domain interaction database to infer IIIs. To confirm with PPI, given an III prediction I_1 _and I_2_, we only keep this prediction if the gene symbols of I_1 _and I_2 _have PPI in IntAct database.

**Table 1:** 19 RNA-seq datasets from SRA

ID	SRA ID	# Exp	Title
**d1**	ERP000546	48	Illumina bodyMap2 transcriptome
**d2**	SRP005169	41	Widespread splicing changes in human brain development and aging
**d3**	SRP005408	31	Gene expression profile in postmortem hippocampus using RNAseq for addicted human samples
**d4**	SRP010280	31	Integrative genome-wide analysis reveals cooperative regulation of alternative splicing by hnRNP proteins
**d5**	SRP002628	30	Comparative transcriptomic analysis of prostate cancer and matched normal tissue using RNA-seq
**d6**	ERP000550	29	Complete transcriptomic landscape of prostate cancer in the Chinese population using RNA-seq
**d7**	SRP005242	21	A Comparison of Single Molecule and Amplification Based Sequencing of Cancer Transcriptomes: RNA-Seq Comparison
**d8**	SRP002079	20	GSE20301: Dynamic transcriptomes during neural differentiation of human embryonic stem cells
**d9**	ERP000992	18	The effect of estrogen and progesterone and their antagonists in Ishikawa cell line compared to MCF7 and T47D cells
**d10**	SRP000727	16	Alternative Isoform Regulation in Human Tissue Transcriptomes
**d11**	SRP007338	16	GSE30017: Widespread regulated alternative splicing of single codons accelerates proteome evolution
**d12**	SRP010166	16	GSE34914: Deep Sequence Analysis of non-small cell lung cancer: Integrated analysis of gene expression, alternative splicing, and single nucleotide variations in lung adenocarcinomas with and without oncogenic KRAS mutations
**d13**	ERP000710	12	Transciptome profiling of ovarian cancer cell lines
**d14**	SRP005411	11	RNA-Seq Quantification of the Complete Transcriptome of Genes Expressed in the Small Airway Epithelium of Nonsmokers and Smokers
**d15**	SRP006731	11	GSE29155: RNA-Seq anlalysis of prostate cancer cell lines using Next Generation Sequencing
**d16**	SRP013224	11	GSE38006: Next-generation sequencing reveals HIV-1-mediated suppression of T cell activation and RNA processing and the regulation of non-coding RNA expression in a CD4+ T cell line
**d17**	ERP000418	10	Gene expression profiles between normal and breast tumor genomes
**d18**	ERP000573	10	RNA and chromatin structure
**d19**	SRP010483	10	GSE35296: The human pancreatic islet transcriptome: impact of pro-inflammatory cytokines

### High-confidence and low-confidence prediction of IIIs

**Figure 3:**
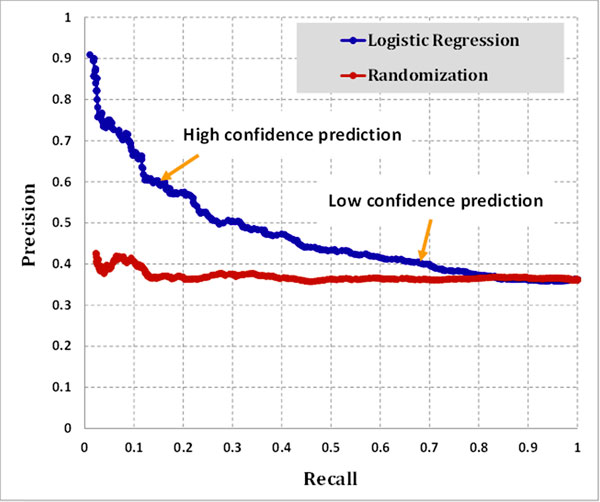
**Precision and recall curve for logistic regression model**. At recall 15%, logistic regression model achieve 60% precision (High-confidence prediction); at recall 68%, logistic regression model achieve 40% precision (Low-confidence prediction).

In the IIIDB, we provided two III prediction sets using the logistic regression model: (a) high-confidence prediction: logit score > 2.575 (precision 60% and recall 15%), it resulted in 4,476 IIIs; and (b) low-confidence prediction: logit score > 1.692 (precision 40% and recall 68%). In addition, given a known PPI, the isoform pair with the best logit score among this PPI will be selected as low-confidence prediction. Thus, a PPI has at least one isoform-isoform interaction. It resulted in 54,605 IIIs (Figure 3).

### Isoform module discovery

To discover functional modules in the III network, we applied MODES network clustering method [16] on low-confidence III network to discover isoform modules with the given parameters (minimum module size 3, maximum module size 30, and density cutoff 0.7). An important feature of MODES is that it can discover overlapping dense isoform modules which allows one isoform to belong to multiple modules. We obtained 1025 modules with size of 5.08 isoforms on average. To provide functional annotation and evaluate the module functional enrichment, we performed the enrichment analyses with GO [17] and KEGG pathway [18] databases. These databases are protein-level annotation which provides approximately functional annotation of isoforms.

To evaluate the significance, we randomly generated the same number and the same size of MODES isoform modules (i.e. 1025 randomized isoform modules). Table 2 shows the results of enrichment analyses for MODES and randomization modules for comparison. The MODES isoform modules have significantly higher functional enrichment rate than those of random cases, showing strong biological relevance of the predicted modules. The MODES isoform modules were further used to build the isoform module database in the IIIDB, and all isoform modules were listed in Additional file 1. We also stored the GO and KEGG enrichment results for all isoform modules in the IIIDB to provide potential functional annotation. In the IIIDB web interface, Figure 1C shows the result page of the isoform module search including the network graph and the enrichment analysis results. In the module result page, users can click any gene symbol to iteratively search isoform modules, and sort the module result table by clicking the column header.

**Table 2:** The enrichment rate of isoform modules based on GO and pathway enrichments

Isoform modules	# modules	% modules enriched with GO^a^	% modules enriched with pathway^b^
MODES modules	1025	88.7%	36.1%
Randomization	1025	49.7%	10.1%

^a ^The modules enriched with GO term (P-value < 0.001).

^b ^The modules enriched with pathway (P-value < 0.01).

### Integrating diverse data sources for III prediction

Currently, most PPI databases do not provide information at the level of isoforms, which thus presents challenges for constructing a gold standard positive set (GSP) for the III prediction. Fortunately, in the June 2013 version of the IntAct database http://www.ebi.ac.uk/intact/[13], we identified 116 human PPIs with isoform specification (out of the total 43,508 distinct human PPIs). For example, IntAct has III between P29590-5 and P03243-1, which correspond to the 5th isoform of the protein P29590 and the 1st isoform of P03243. In addition, to obtain more IIIs for the GSP, we applied the following rule: given a PPI between protein P1 and P2, if both P1 and P2 only have single isoforms we also take it as the GSP. It resulted in 11,356 IIIs in the GSP set.

GSP covered 5,503 RefSeq IDs, and we used these RefSeq IDs to construct gold standard negative set (GSN). The GSN was defined as isoform pairs in which one isoform was assigned to the plasma membrane cellular component, and the other was assigned to the nuclear cellular component by the isoform-specific sub-cellular localization, in which we performed sequence-based predictions using the CELLO (subCELlular LOcalization predictor) [19]. To obtain the accurate isoform-specific annotation, we only used the cellular localization prediction results that consist of UniProt GO annotations. It resulted in 36 RefSeq IDs for plasma membrane cellular component and 739 RefSeq IDs for nuclear cellular component. In addition, isoforms that are assigned to both the plasma membrane and the nuclear cellular component are excluded in GSN.

To calculate precision and recall, we used timestamp to divided GSP into training and test GSP sets, in which if an interaction with timestamp after 1st Jan 2012, it will be assigned to test GSP set (10,408 IIIs); otherwise, it will be assign to training GSP set (948 IIIs). When the GSP is decided, we used the RefSeq IDs covered in GSP to build GSN. Figure 3 shows the precision and recall curve for the logistic regression model.

### Case studies

To demonstrate the biological importance of the IIIDB, we searched for isoform-associated reports in the literature. Although isoform-specific protein function studies are very rare, we found isoform-specific biological evidences with BCL2L1, which validated our III prediction of BCL2L1. In addition, we also found diverse biological functions with Ras association domain family in our isoform modules.

#### BCL2L1

BCL2L1 has two isoforms (Table 3), in which BCL2L1 isoform 1 is called Bcl-xL (long form) and BCL2L1 isoform 2 is called Bcl-xS (short form) [3]. These isoforms play important roles in apoptosis as follows: Bcl-xL inhibits apoptosis whereas Bcl-xS promotes apoptosis [4]. In our high-confidence prediction, BCL2L1 isoform 1 (Bcl-xL) interacts with BAK1, BAX and NLRP1 to inhibit apoptosis, but BCL2L1 isoform 2 (Bcl-xS) doesn't (Table 3). In previous reports, the fluorescence anisotropy, analytical ultracentrifugation, and NMR assays confirmed a direct interaction between Bcl-xL and BAK1 [5,20]. Vogler et al. also reported that the interaction of Bcl-xL and BAK1 in platelets ensures cell survival [5]. Edlich *et al*. reported that an interaction between Bcl-xL and BAX not only inhibits BAX activity but also maintains BAX in the cytosol [21]. On the other hand, Chang et al. demonstrated that Bcl-xL interacted with endogenous BAX in 293 cells. However, no significant amount of BAX was detectable in the Bcl-xS immunoprecipitation [21], suggesting that Bcl-xS does not interact with BAX. In addition, Bruey et al. reported that Bcl-xL interacts with NALP1 to suppress apoptosis [22]. Thus, these previous biological studies validated our III prediction of BCL2L1.

**Table 3:** BCL2L1 isoform interaction partners.

Isoform	RefSeq ID	mRNA length	Protein length	UniProt ID	Interaction partner (high confidence)
**BCL2L1 isoform 1 (Bcl-xL)**	NM_138578	2575	233	Q07817-1	BAK1 BAX NLRP1
**BCL2L1 isoform 2 (Bcl-xS)**	NM_001191	2386	170	Q07817-2	BCL2

#### RASSF1

**Figure 4:**
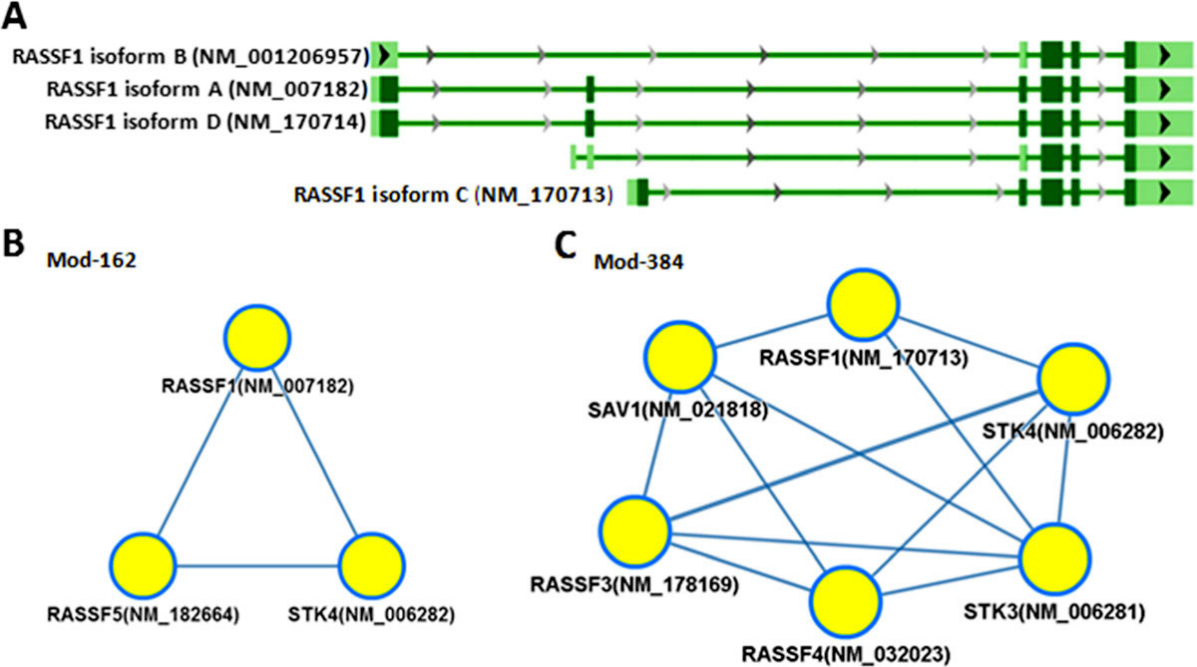
**Two isoform modules of RASSF1**. **(A) **RASSF1 has four isoforms (isoform A, B, C and D), of which two isoforms belong to module Mod-162 and Mod-384. **(B) **The isoform module Mod-162 includes RASSF1 isoform A (NM_007182), RASSF5 and STK4. **(C) **The isoform module Mod-384 includes RASSF1 isoform C (NM_170713), RASSF3, RASSF4, SAV1, STK3 and STK4.

RASSF1 is RAS association domain family member 1. RASSF1 has four isoforms, of which two isoforms belong to isoform modules (Mod-162 and Mod-384). The Mod-162 consists of RASSF1 isoform A, RASSF5 and STK4, whereas the Mod-384 consists of RASSF1 isoform C, RASSF3, RASSF4, SAV1, STK3 and STK4 (Figure 4). Interestingly, several studies reported that RASSF5 may interact with RASSF1 isoform A to suppress tumors [23-25], suggesting that the Mod-162 is validated. On the other hand, RASSF1 isoform C may play a completely different role as an oncogene in high-grade tumors [26]. Although the function of RASSF1 isoform C is still not clear, the RASSF1 isoform A and C should have distinct functions. The IIIDB assigned the RASSF1 isoform A and C into the Mod-162 and the Mod-384, respectively, suggesting a new hypothesis of the isoform-level modules.

## Methods

### Isoform coexpression network construction

To construct the Isoform coexpression networks, we selected 19 RNA-seq datasets with at least 10 experiments from Sequence Read Archive (SRA) database (Table 1) [27]. We performed the eXpress [28] with Bowtie2 aligner [29] to obtain isoform expression values. NCBI Reference Sequences (RefSeq) mRNAs with protein sequences was used as transcriptome annotation, which included 31,454 RefSeq IDs (Jan 2013 version) [15].

### Logistic regression model

The logistic regression model has been applied to PPI prediction [30,31], as it is suitable to describe the relationship between a binary response variable and a set of explanatory variables. We used logistic regression approach to build the prediction model as follows:

$$logit\left(y_{ij}\right)=\alpha_{0}+\alpha_{1}E1_{ij}+\alpha_{2}E2_{ij}+\cdots +\alpha_{19}E19_{ij}+\alpha_{20}DDI_{ij}$$

where *α*_0_,*α*_1_,...,*α*_20 _are regression coefficients and *y_ij _*is the probability of the isoform interaction between isoform *i *and isoform *j. DDI, E1, E2, ..., E19 *are described as follows: **(a) *Domain-domain interaction (DDI) score*: **For all combinations of human isoform pairs, if a isoform pair has domain-domain interaction in the DOMINE database [32,33], then we assign the DDI score by the confidence level in the DOMINE database as follows: high-confidence prediction: 3, medium-confidence prediction: 2, and low-confidence prediction: 1. If the isoform pair has several DDI scores, we take the highest score. **(b) *19 RNA-seq datasets (E1, E2, ..., E19)*: **the absolute values of Pearson correlations for all isoform pairs derived from RNA-seq datasets. Since each RNA-seq dataset may have different quality and data type, we used the logistic regression model to integrate 19 RNA-seq datasets, and then the coefficients of datasets reflected quality of the RNA-seq datasets.

## Competing interests

The authors declare that they have no competing interests.

## Supplementary Material

Additional file 1 **The isoform modules**. There are 1025 isoform modules with gene symbols and RefSeq IDs. Click here for file
